# Probing the Interplay of Protein Self‐Assembly and Covalent Bond Formation in Photo‐Crosslinked Silk Fibroin Hydrogels

**DOI:** 10.1002/smll.202407923

**Published:** 2024-11-16

**Authors:** Hien A. Tran, Anton Maraldo, Trinh Thi‐Phuong Ho, Mai Thanh Thai, Quinn van Hilst, Habib Joukhdar, Marija Kordanovski, Jugal Kishore Sahoo, Onur Hartsuk, Miguel Santos, Steven G. Wise, David L. Kaplan, Thanh Nho Do, Kristopher A. Kilian, Khoon S. Lim, Jelena Rnjak‐Kovacina

**Affiliations:** ^1^ Graduate School of Biomedical Engineering University of New South Wales Sydney NSW 2052 Australia; ^2^ College of Engineering & Computer Science and VinUni‐Illinois Smart Health Center Hanoi 100000 Vietnam; ^3^ Chronic Diseases Theme School of Medical Sciences University of Sydney Sydney NSW 2006 Australia; ^4^ Department of Biomedical Engineering Tufts University Boston MA 02155 USA; ^5^ School of Chemistry University of New South Wales Sydney NSW 2052 Australia; ^6^ Australian Center for Nanomedicine University of New South Wales Sydney NSW 2052 Australia; ^7^ School of Materials Science and Engineering University of New South Wales Sydney Sydney NSW 2052 Australia; ^8^ School of Clinical Medicine Faculty of Medicine and Health University of New South Wales Sydney NSW 2052 Australia; ^9^ Tyree Foundation Institute of Health Engineering Sydney NSW 2052 Australia

**Keywords:** beta‐sheet transition, di‐tyrosine, hydrogels, photo‐crosslinking, self‐assembly, silk

## Abstract

Covalent crosslinking of silk fibroin via native tyrosine residues has been extensively explored; however, while these materials are very promising for biomedical, optical, soft robotics, and sensor applications, their structure and mechanical properties are unstable over time. This instability results in spontaneous silk self‐assembly and stiffening over time, a process that is poorly understood. This study investigates the interplay between self‐assembly and di‐tyrosine bond formation in silk hydrogels photo‐crosslinked using ruthenium (Ru) and sodium persulfate (SPS) with visible light. The effects of silk concentration, molecular weight, Ru/SPS concentration, and solvent conditions are examined. The Ru/SPS system enables rapid crosslinking, achieving gelation within seconds and incorporating over 90% of silk into the network, even at very low protein concentrations (≥0.75% wt/v). A model emerges where silk self‐assembly both before and after crosslinking affects protein phase separation, mesoscale structure, and dynamic changes in the hydrogel network over time. Silk concentration has the greatest impact on hydrogel properties, with higher silk concentration hydrogels experiencing two orders of magnitude increase in stiffness within 1 week. This new understanding and ability to tune hydrogel properties and dynamic stiffening aids in developing advanced materials for 4D biofabrication, sensing, 3D cancer models, drug delivery, and soft robotics.

## Graphical abstract

This study explores the interplay between self‐assembly and di‐tyrosine bond formation in silk fibroin hydrogels photo‐crosslinked with ruthenium and sodium persulfate. The resultant dual crosslinked network is dynamic and changes over time, with hydrogel morphology, mechanical properties, and opacity tailorable via silk concentration, molecular weight, and crosslinker concentration for a range of biomedical applications.

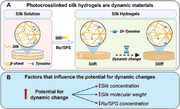

## 1 Introduction

Covalent crosslinking of silk fibroin using its native tyrosine amino acids (comprising 5.3% of total residue count) has gained popularity for producing transparent, elastomeric silk hydrogels.^[^ ^1^, ^2^, ^3^, ^4^ ^]^ This stands in contrast with the opaque and brittle hydrogels formed through the physical crosslinking of silk, specifically via the formation of beta‐sheets.^[^ ^5^, ^6^, ^7^ ^]^ While much of the existing literature employs horseradish peroxidase (HRP) and hydrogen peroxide (H_2_O_2_) to catalyze the creation of di‐tyrosine bonds, alternative methods, such as Ru and SPS based photoinitiated crosslinking, have also been explored.^[^ ^1^, ^8^, ^9^, ^10^ ^]^ Intriguingly, despite establishing covalent di‐tyrosine bonds, the silk chains in these hydrogels still exhibit dynamic behavior post‐crosslinking through a self‐assembly process that rearranges the silk structure to form beta‐sheets.^[^ ^11^, ^12^, ^13^ ^]^ This dynamic process results in hydrogels possessing both covalent and physical crosslinks^[^ ^11^ ^]^ and leads to hydrogel stiffening and opacity over time.

This dynamic behavior presents an opportunity to investigate silk self‐assembly mechanics, model tissue stiffening associated with various diseases, and study cellular responses to dynamic microenvironments.^[^ ^14^, ^15^ ^]^ However, this dynamic aspect may be undesirable in certain applications requiring static conditions, and our limited understanding of this process hinders our ability to regulate it effectively.

Our previous research demonstrated significant differences in the kinetics of dynamic changes between silk crosslinked using HRP/H_2_O_2_ and Ru/SPS, despite both systems catalyzing the formation of di‐tyrosine bonds and having similar crosslinking density.^[^ ^8^ ^]^ Notably, HRP/H_2_O_2_ hydrogels rapidly stiffened over a 4‐week incubation in phosphate buffered saline (PBS), while Ru/SPS hydrogels did not.^[^ ^8^ ^]^ This discrepancy suggests that the kinetics of the crosslinking reaction play a crucial role. The HRP reaction takes ≈30 min to crosslink silk,^[^ ^8^, ^16^ ^]^ whereas the Ru/SPS reaction occurs in just 3 min, with gelation onset at ≈30 s.^[^ ^8^ ^]^ This implies that changes in silk occur both before and during crosslinking, influencing the downstream dynamic behavior of the resultant hydrogels. However, these dynamic changes have only been studied in 2% wt/wt silk, and the impact of silk self‐assembly and molecular weight properties, crosslinking conditions, and the mechanisms underlying these dynamic changes remain poorly understood.^[^ ^8^ ^]^

In this study, we aim to understand the dynamic behavior of silk fibroin during and after the formation of di‐tyrosine crosslinks. We investigate the gelation behavior and properties of Ru/SPS‐crosslinked silk hydrogels produced with varying silk fibroin concentrations, molecular weights, Ru/SPS concentrations, and solvent conditions. This exploration carries significant implications for understanding the role of tyrosine moieties and di‐tyrosine bonds in self‐assembly,^[^ ^17^ ^]^ and developing covalently induced silk hydrogels with tuneable and controllable physical properties for applications in 4D biofabrication, sensing technologies, in vitro 3D cancer models, targeted drug delivery systems, and soft robotics.

## 2 Results and Discussion

To study the interplay between self‐assembly and di‐tyrosine bond formation in Ru/SPS silk hydrogels, we investigated the effect of silk concentration and molecular weight, Ru/SPS concentration, and solvent conditions on hydrogel properties. Silk molecular weight was controlled via silk fiber degumming time, resulting in polydisperse silk populations, with longer degumming time resulting in smaller molecular weight protein species.^[^ ^18^, ^19^ ^]^ Here, they are referred to as high (10‐min degumming), medium (30‐min degumming), and low (60‐min degumming) molecular weight.

### 2.1 Silk Hydrogel Manufacture via Ru/SPS Catalyzed Di‐Tyrosine Bond Formation

**Figure 1:**
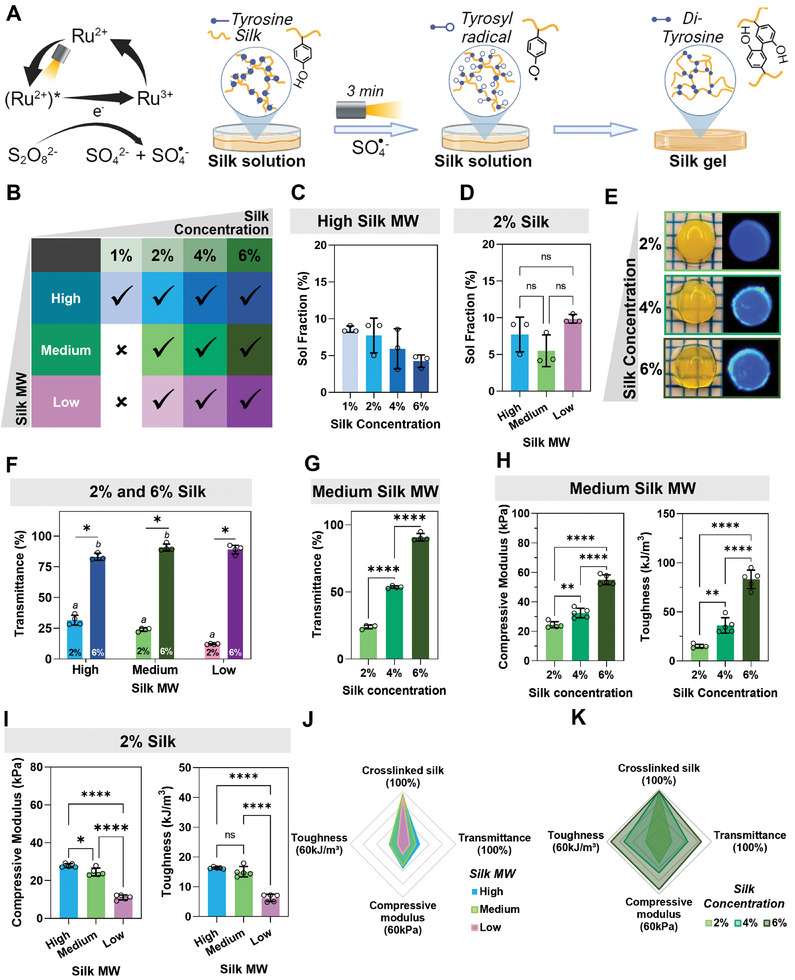
Silk hydrogels with tuneable physical properties formed via the Ru/SPS photo‐crosslinking reaction. A) Schematic summary of the Ru/SPS crosslinking reaction in unmodified silk fibroin where, in the presence of visible light, tyrosine residues are oxidized to tyrosyl radicals that undergo coupling to form di‐tyrosine bonds, resulting in the formation of silk hydrogels. B) Summary of the Ru/SPS crosslinking efficacy at different silk concentrations (1–6% wt/v) and molecular weights (low, medium, high). Ticks indicate hydrogel formation. C) Sol fraction of high molecular weight silk (10‐min boiled) at different silk concentrations showing that over 90% of silk was incorporated into the hydrogel network regardless of the silk concentration. D) Sol fraction of 2% silk hydrogels at different molecular weights. E) Representative images of silk hydrogels at different concentrations with corresponding autofluorescence associated with di‐tyrosine bonds. F) Light transmittance through 2% and 6% silk hydrogels made from different molecular weight silk. ^a^ or ^b^ show significant differences between hydrogels made from different molecular weight silks at 2% or 6%, respectively. G) Light transmittance through silk hydrogels made from medium molecular weight (30 min boiled) silk at different protein concentrations. H) Compressive modulus and toughness of silk hydrogels made from medium molecular weight (30 min boiled) silk at different protein concentrations. I) The compressive modulus and toughness of 2% silk hydrogels made from different molecular weight silks. J,K) Summary graphs showing the tuneable properties of silk hydrogels by changing silk molecular weight (J) or silk concentration (K). All data are mean ± SD, *n* = 3–4. a,b,*p < 0.05, **p < 0.01, ****p < 0.0001, ns or no indications = no significant difference.

In the Ru/SPS crosslinking system (**Figure** **1**A), Ru undergoes photolysis when exposed to visible light by donating electrons to a persulfate compound (SPS), generating an excited Ru^3+^ species that can oxidize aromatic residues, particularly tyrosine. The oxidized tyrosine residues are transformed into tyrosyl radicals, which can then undergo coupling to form di‐tyrosine bonds. Due to its straightforward and versatile nature, this reaction has gained popularity for crosslinking tyrosine‐rich natural polymers like gelatin, resilin, fibrinogen, and silk.^[^ ^2^, ^20^ ^]^ This reaction is particularly advantageous, as it does not require chemical modification of the silk protein to generate hydrogels.

The commonly reported Ru/SPS concentration for silk crosslinking in the literature is 0.5 mm Ru and 5 mm SPS.^[^ ^3^, ^8^, ^21^ ^]^ Consequently, we adopted this concentration to investigate silk gelation at various concentrations and molecular weights. Silk formed hydrogels at 2% wt/v and above, regardless of its molecular weight. However, at 1% wt/v silk, only high molecular weight (10‐min deguming) silk formed a hydrogel (Figure 1B). While previous studies have indicated a slightly higher tyrosine content in high molecular weight silk (5.3% at 5‐min degumming vs 5.2% at 30‐min and 60‐min degumming),^[^ ^19^ ^]^ it is more likely that the proximity of tyrosines to each other in the longer silk chains contributed to effective crosslinking at 1% wt/v silk, rather than the slightly higher tyrosine content.

In general, the Ru/SPS crosslinking reaction was efficient, resulting in a 4–10% sol fraction, indicating that 90–96% of the protein was successfully incorporated into the hydrogel network (Figure 1C,D), consistent with other reports of Ru/SPS crosslinking of silk.^[^ ^8^ ^]^ Moreover, the formed hydrogels exhibited the typical fluorescence in the UV range associated with di‐tyrosine bonds (Figures 1E and S1, Supporting Information),^[^ ^22^, ^23^ ^]^ confirming covalent bond formation.

Interestingly, light transmittance through silk hydrogels was dependent on silk fibroin concentration, where transmittance increases with higher silk concentrations, as illustrated in Figure 1F. Specifically, 6% hydrogels supported over 90% light transmittance, while 2% wt/v hydrogels only supported ≈23%. At low silk concentrations (2% wt/v), light transmittance also varied with silk molecular weight, decreasing with increasing molecular weight. However, this trend was not observed for silk with a high concentration (6%) (Figure 1G).

Mechanical properties of the silk hydrogels tested under compression followed the expected pattern, with compressive modulus and toughness increasing with higher silk concentration (Figures 1H and S2A, Supporting Information) and molecular weight (Figures 1I and S2B, Supporting Information), as previously reported for multiple silk material formats.^[^ ^18^, ^24^, ^25^ ^]^ In this system, the compressive modulus was modulated between ≈6 and 84 kPa by adjusting the silk concentration (1–6% wt/v) and molecular weight (10‐min–60‐min degumming).

These findings collectively demonstrate that silk concentration and molecular weight play significant roles in silk hydrogel formation using the Ru/SPS crosslinking method, resulting in hydrogels with tuneable physical properties especially via silk concentration, as summarized in Figure 1J,K.

### 2.2 Silk Concentration Affects Polymer Network Assembly Pre‐ and Post‐Crosslinking

**Figure 2:**
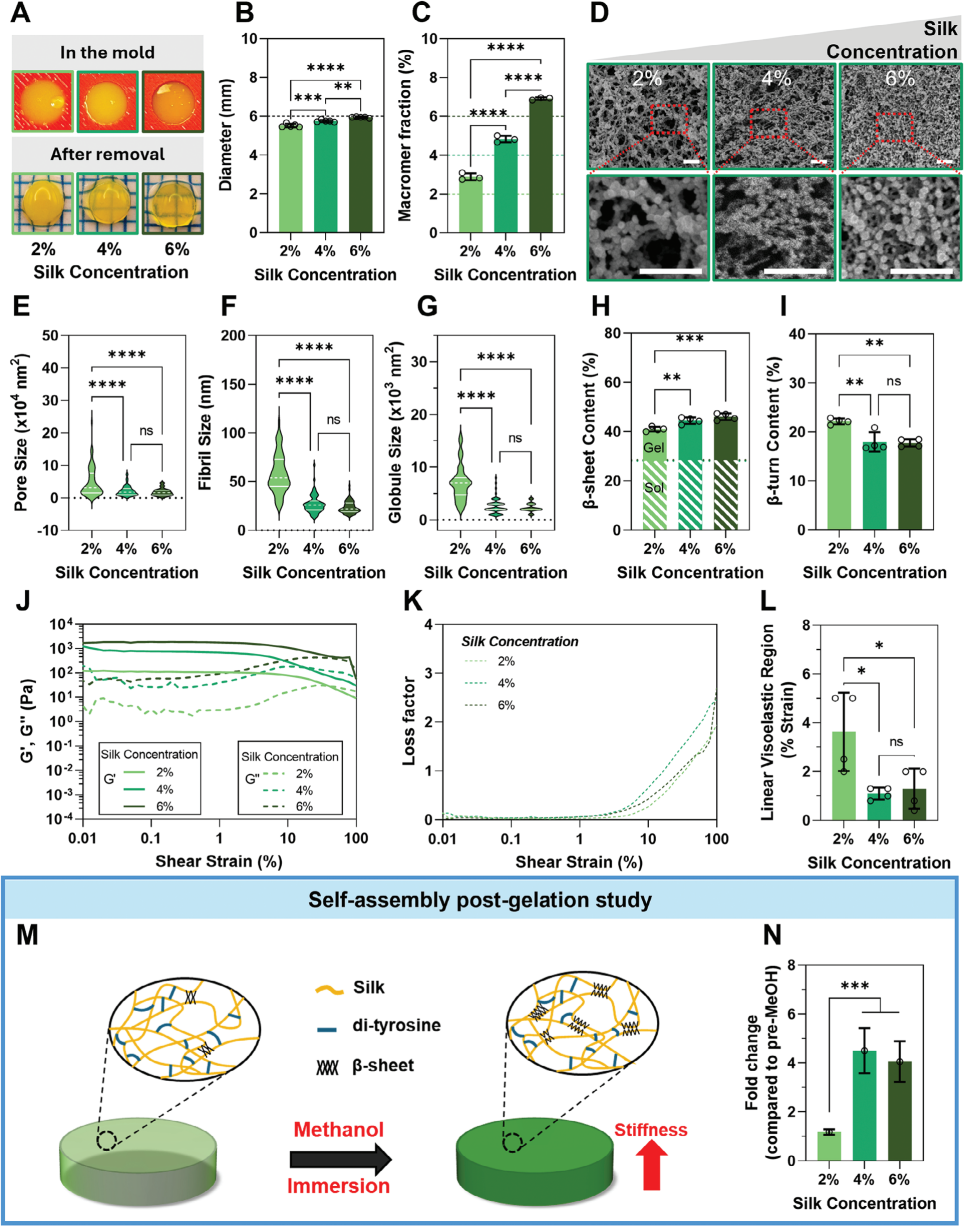
Silk concentration regulates self‐assembly pre‐ and post‐crosslinking. A–C) Silk hydrogels at low concentration (2%) post‐crosslinking expelled water after being removed from the mold (A), resulting in smaller diameter hydrogels than the mold (B) and a higher macromer fraction (C). The black dashed line indicates the mold size (B), and the white‐green, green, and dark green dashed lines indicate the expected macromer fraction of 2%, 4%, and 6% silk, respectively (C). D) Representative SEM images of silk hydrogels at different silk concentrations. White scale bars are 500 nm. Hydrogel network morphology was quantified as E) pore size, F) fibril size, and G) globule size from the SEM images. *N* = 3–4 samples with at least 18 counts per image, *n* ≥ 54. Data was collected through a randomized process at the intersections of a grid overlayed over each image. H,I) Quantitative analysis of ATR‐FTIR of Amide I region showing high beta‐sheet content (H) and low beta‐turn content (I) at different silk concentrations. The white dashed region indicates the beta‐sheet content of the silk solution prior to crosslinking at different silk concentrations. J–L) Rheological analysis of silk hydrogels showing the storage and loss modulus (J), and loss factor (K), and linear viscoelasticity region (L) against the shear on silk concentrations and self‐assembly pre‐crosslinking. M) Schematic summary showing expected changes in silk secondary structure (β‐sheet formation) and mechanical properties (increase in stiffness) following incubation in methanol. N) Fold change in compressive modulus of silk hydrogels after incubation in methanol (MeOH) compared to pre‐MeOH. All data are mean ± SD, *n* = 3–4. *p < 0.05, **p < 0.01, ****p < 0.0001, ns or no indications = no significant difference.

Even though under standard conditions (30‐min degumming silk, 0.5/5 mm Ru/SPS), silk successfully formed stable, robust hydrogels within a concentration range of 2–6% wt/v, it was interesting to observe that as the silk concentration increased, differences in hydrogel diameter and transmittance emerged. Low‐concentration hydrogels had smaller diameters (5.5 mm at 2% hydrogels) than the original mold (6 mm), apparently resulting in more tightly packed polymer networks (**Figure** **2**A,B). This phenomenon, previously noted in other polymers, involves water expulsion during crosslinking, leading to a denser polymer network.^[^ ^26^, ^27^, ^28^ ^]^ To investigate this, the macromer fraction for each silk concentration was assessed (Figure 2C). In theory, the macromer fraction should align with the initial silk concentration. However, for each condition, it exceeded the anticipated value, suggesting that water expulsion during crosslinking increased the silk concentration in the final hydrogels compared to the initial pre‐gelled solution. Relative to the initial concentration, the highest increase in macromer fraction and, thus, hydrogel densification was in 2% hydrogels (≈1.45‐fold relative to ≈1.15‐fold in 6% hydrogels).

SEM analysis (Figure 2D) of the as‐formed hydrogels (before swelling) demonstrated distinct differences in the polymer network, with 2% hydrogels exhibiting a more heterogeneous network relative to 4% and, especially, 6% hydrogels. This disparity in hydrogel homogeneity likely explains the differences in light transmittance through the hydrogels, as a more homogeneous network is less prone to light scattering, resulting in higher transmittance. The hydrogel network comprised nanoscale spherical structures that coalesced into a porous fibrous network, aligning with current models of protein self‐assembly.^[^ ^29^, ^30^, ^31^, ^32^ ^]^ Similar nano‐scale spheres that coalesce into mesoscale structures were observed in other self‐assembling proteins, including tropoelastin and α‐synuclein and are the result of native and functional phase separation.^[^ ^33^, ^34^ ^]^ In 2% hydrogels, the spheres and resultant fibers and pores were larger compared to 6% hydrogels. Additionally, in 6% hydrogels, fibers seemed to form from single spheres coalescing into thin fibers, while in 2% hydrogels, the spheres appeared to coalesce into larger sheet‐like lamellae (Figure 2E–G).

This data indicates that self‐assembly varied across different silk concentrations, leading to distinct morphologies that become fixed during Ru/SPS crosslinking. Analysis of beta‐sheet content, a marker of silk self‐assembly, showed higher overall beta‐sheet content in 4% and 6% hydrogels, relative to 2% hydrogels. Furthermore, compared with the beta‐sheet content in silk solutions, 4% and 6% hydrogels had a higher increase in beta‐sheet content (Figures 2H and S3A, Supporting Information) and lower beta‐turn content (Figure 2I), demonstrating that silk concentration affected silk protein self‐assembly in the crosslinked form. It is plausible that di‐tyrosine bond formation brought silk chains closer to each other, allowing for more effective self‐assembly.^[^ ^35^ ^]^ Rheological analysis showed that increasing silk concentration increased the stiffness and toughness but reduced the elasticity of the hydrogel network (Figure 2J). The large sheet‐like lamellar fibers at low silk concentration hydrogels (2%) are likely weaker than a dense network of thin fibers at high silk concentration hydrogels (6%), yielding 90.24 Pa and 1.6 kPa of shear stress, respectively. However, the yield strain decreased with increasing silk concentration (≈2‐fold reduction), resulting in the reduction of the linear viscoelastic region (Figure 2K,L), showing that the dense network of thin fibers was more rigid and fixed than large sheet‐like lamellar fibers against shear thinning stress. This result correlated with the beta‐sheet content, which was higher in 4% and 6% hydrogels.

The differences observed in the structure of hydrogels, both in terms of β‐sheet formation and liquid–liquid phase separation that results in the formation of globules and fibrils,^[^ ^32^, ^34^ ^]^ at different silk concentrations are expected to affect the consequent dynamic changes in silk self‐assembly and properties over time. To explore how these differences affect the potential for beta‐sheet formation and hydrogel stiffening after crosslinking, hydrogels were incubated in methanol, which is well‐documented to force high beta‐sheet formation in silk (Figure 2M), and their compressive modulus (more beta sheets = stiffer hydrogels) was measured.^[^ ^1^, ^13^, ^36^, ^37^ ^]^ The increase in compressive modulus following methanol incubation (Figure 2N) was significantly higher in 4% and 6% hydrogels relative to 2% hydrogels, suggesting they have a higher propensity for dynamic stiffening after di‐tyrosine crosslinking.

### 2.3 Silk Molecular Weight Affects Polymer Network Assembly Pre‐ and Post‐Crosslinking

After observing the impact of silk concentration on the polymer network self‐assembly and the potential for dynamic changes, we investigated the influence of silk molecular weight on these parameters. Silk hydrogels were made using 2% wt/v silk and 0.5/5 mm Ru/SPS with varying molecular weights by controlling the degumming time (10‐min degumming‐high, 30‐min degumming‐medium, 60‐min degumming‐low).

**Figure 3:**
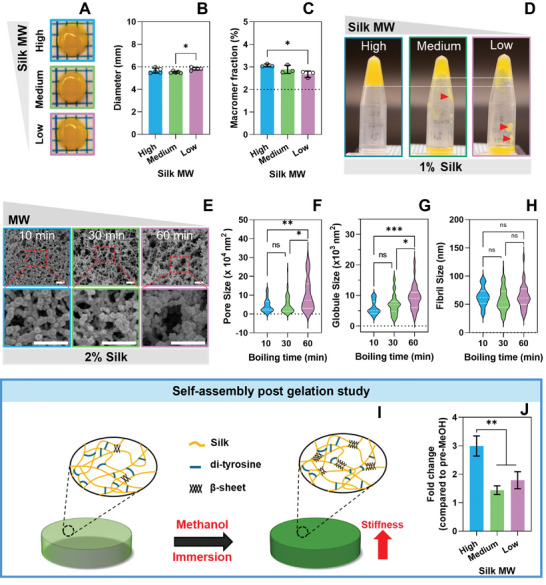
The effect of silk molecular weight on the self‐assembly of silk hydrogels. A) Representative images of silk hydrogels and B) quantitative analysis of the hydrogel diameter at different molecular weights. The dashed black line indicates the diameter of the mould. C) Macromer fraction of silk hydrogels at different molecular weights. The dashed line indicates the initial silk concentration. D) Representative images of flipped tubes showing the crosslinking efficacy of different molecular weight silks at low silk concentration (1% wt/v). The dashed white line indicates the level of formed hydrogels, while red arrows indicate inconsistent crosslinking. E) Representative SEM images of silk hydrogels at different molecular weights. White scale bar are 500 nm. Hydrogel network morphology was quantified as F) pore size, G) globule size, and H) fibril size, *N* = 3–4 with at least 10 counts per image, *n* ≥ 30. Data was collected through a randomized process at the intersection of a grid overlayed over each image. I) Schematic summary showing expected changes in silk secondary structure (β‐sheet formation) and mechanical properties (increase in stiffness) following incubation in methanol J) Fold change in compressive modulus of silk hydrogels after incubation in methanol (MeOH) compared to pre‐MeOH at different molecular weights. All data are mean ± SD, *n* = 3–4. *p < 0.05, **p < 0.01, ****p < 0.0001, ns or no indication = no significant difference.

During crosslinking, all hydrogels expelled water, resulting in diameters slightly smaller than the original mold (**Figure** **3**A,B), and the macromer fraction exceeded the original 2%, indicating network densification (Figure 3B). This effect was more pronounced in high molecular weight (10‐min degumming) silk, showing a significantly higher macromer fraction than in low molecular weight (60‐min degumming) silk. This phenomenon may explain the feasibility of obtaining hydrogels from very low concentration (even at 0.75%wt/v) in 10‐min degumming silk, where the network contracts and allows crosslink formation despite the low protein concentration (Figures 3D and S4, Supporting Information).

Interestingly, the high molecular weight silk exhibited a similar network morphology to the medium molecular weight silk (Figure 3E), confirmed through quantitative analysis of sphere, fiber, and pore sizes in SEM images (Figure 3F–H), which were not significantly different between high and medium molecular weight silk. In contrast, low molecular weight silk displayed an even more pronounced sheet‐like morphology, where spheres coalesced into flat structures and formed fewer, larger pores. Hence, low silk concentration and low molecular weight produce more sheet‐like lamellar than fibrous morphologies in the hydrogel network.

Like observations with silk concentration, the molecular weight affected the propensity for dynamic changes of hydrogels post‐crosslinking (Figure 3I,J). The high molecular weight hydrogels showed a high propensity for dynamic changes, similar to high silk concentration (6% wt/v).

### 2.4 The Effect of Ions on Silk‐Self‐Assembly and Dynamic Hydrogel Changes

Our investigations into silk concentration and molecular weight indicate that these properties affect dynamic changes in silk hydrogels over time, but the studies were conducted on hydrogels prepared in water and silk self‐assembly post‐crosslinking was forced through immersion in methanol, an environment that artificially induces beta‐sheet formation. To understand silk self‐assembly following di‐tyrosine crosslinking better, we studied the effect of ions on silk hydrogel formation and on the dynamic changes in the silk structure over time in physiological buffers.

**Figure 4:**
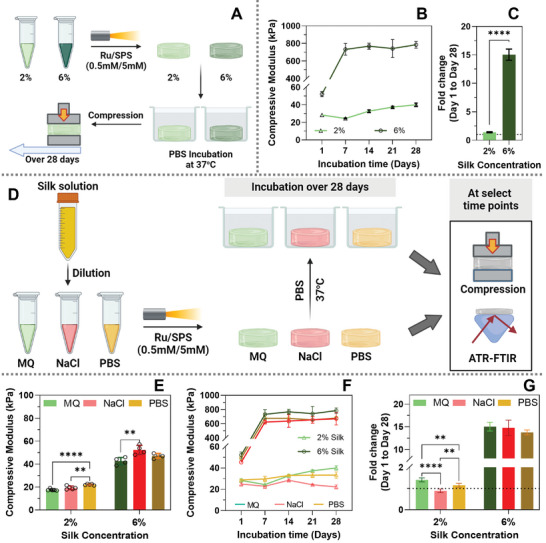
Ion interactions regulate the self‐assembly of silk hydrogels. A) Schematic representation of the experiments to study the dynamic changes in silk hydrogels over time. Silk was diluted to 2% and 6% in ion‐free water and crosslinked with Ru/SPS. Hydrogels were then incubated in PBS, and the compressive modulus was determined at multiple timepoints in the 28‐day incubation period. B) Compressive modulus of 2% and 6% silk hydrogels incubated in PBS over a 28‐day period. C) Fold‐change in compressive modulus after 28 days in PBS relative to day 1. D) Schematic representation of the experiments to study the effect of ions in the silk solution prior to crosslinking on the dynamic changes in silk hydrogels over time. Silk was diluted in ion‐free water, 0.9% w/v NaCl or PBS to 2% or 6% and crosslinked with Ru/SPS. Hydrogels were then incubated in PBS and the compressive modulus was determined at multiple time points in the 28‐day incubation period. E) Changes in compressive modulus of 2% and 6% silk hydrogels in different solvents of as‐made hydrogels. F) Compressive modulus over 28 days and (G) Fold‐change in compressive modulus at day 28 relative to day 1. All data are mean of at least four replicates ± SD. * indicated the significant differences between solvents within one time point, while ^#^ indicated the significant differences between day 1 and day 28 within each solvent. *p < 0.05, **p < 0.01, ****p < 0.0001, ns or no indications = no significant difference.

Initially, we examined the effect of silk concentration on the dynamic stiffening of silk hydrogels by measuring the stiffness of 2% and 6% hydrogels when incubated in PBS at 37 °C over a 4‐week period (**Figure** **4**A). Silk was diluted in water as the solvent, and the hydrogels were crosslinked with 0.5 mm/5 mm Ru/SPS before incubation in PBS. The 2% hydrogels gradually stiffened over time, with the compressive modulus increasing from 28.3 to 40 kPa, a 1.4‐fold increase over 4 weeks (Figure 4B,C). In contrast, the 6% hydrogels rapidly increased from 52 to 732 kPa within the first week and remained stable, resulting in an overall ≈15‐fold increase over 4 weeks (Figure 4B,C).

Ions are known to affect the self‐assembly of a variety of proteins, including silk, where ions interact with hydrated surfaces of the backbone and side chains, influencing their conformational stability and assembly behavior.^[^ ^38^, ^39^ ^]^ For example, the presence of sodium ions (Na^+^) was previously shown to enhance the stability of silk against thermal and chemical denaturation,^[^ ^40^ ^]^ and promote self‐assembly to form hydrogels.^[^ ^41^ ^]^ Moreover, potassium ions (K^+^) induced phase separation in silk, resulting in the formation of particles/spheres,^[^ ^42^ ^]^ while Ca^2+^ions are known to form ionic bonds with COO^−^ ions of amino acid side chains leading to the formation of clusters.^[^ ^25^ ^]^ Recently, anions were also shown to affect silk self‐assembly.^[^ ^43^ ^]^

In light of this, we explored the effect of the silk solvent on the dynamic stiffening of silk hydrogels by diluting silk in ion‐free water, NaCl, or PBS (which contains Na^+^ and HPO_4_^2−^ ions known to promote protein salting out or aggregation) (Figure 4D).^[^ ^38^, ^44^, ^45^ ^]^ The mechanical properties of the resulting hydrogels were influenced by the solvent, with hydrogels made in NaCl and PBS displaying a higher compressive modulus compared to those formed in water (Figure 4E) in both 2% and 6% silk hydrogels. This suggests that ions in those solvents enhance the initial silk self‐assembly before crosslinking, resulting in stiffer and tougher hydrogels. The results were further supported by the higher beta‐sheet content observed in hydrogels made in NaCl and PBS relative to those in ion‐free water (Figure S5, Supporting Information).

Hydrogels prepared in NaCl and PBS showed less stiffening than those made in ion‐free water, with the effect more pronounced in 2% hydrogels relative to 6% hydrogels (Figure 4E–G). Notably, 2% hydrogels made in NaCl showed no change in stiffness over the 4 week period (Figure 4F,G), consistent with previous studies comparing 2% wt/v silk in NaCl crosslinked with Ru/SPS versus HRP/H_2_O_2_, in which photo‐induced hydrogels remained stable while enzymatically induced hydrogels underwent rapid stiffening.^[^ ^8^ ^]^ At 6% silk, all hydrogels made in ion‐free water, NaCl, and PBS showed a high degree of stiffening after one week of incubation and remained stable over the rest of the experiment period. It is important to note that the effect of hydrogel stiffening might not only be due to the formation of beta‐sheets as 6% hydrogels made in PBS showed no significant difference in beta‐sheet content at day 28 (40.54 ± 1.6%) compared to day 1 (39.47 ± 0.3%) (Figure S5, Supporting Information), and these potential ionic interactions are of interest in future studies.

### 2.5 Ruthenium/SPS Catalyzes Di‐Tyrosine Bond Formation and Affects Silk Self‐Assembly

**Figure 5:**
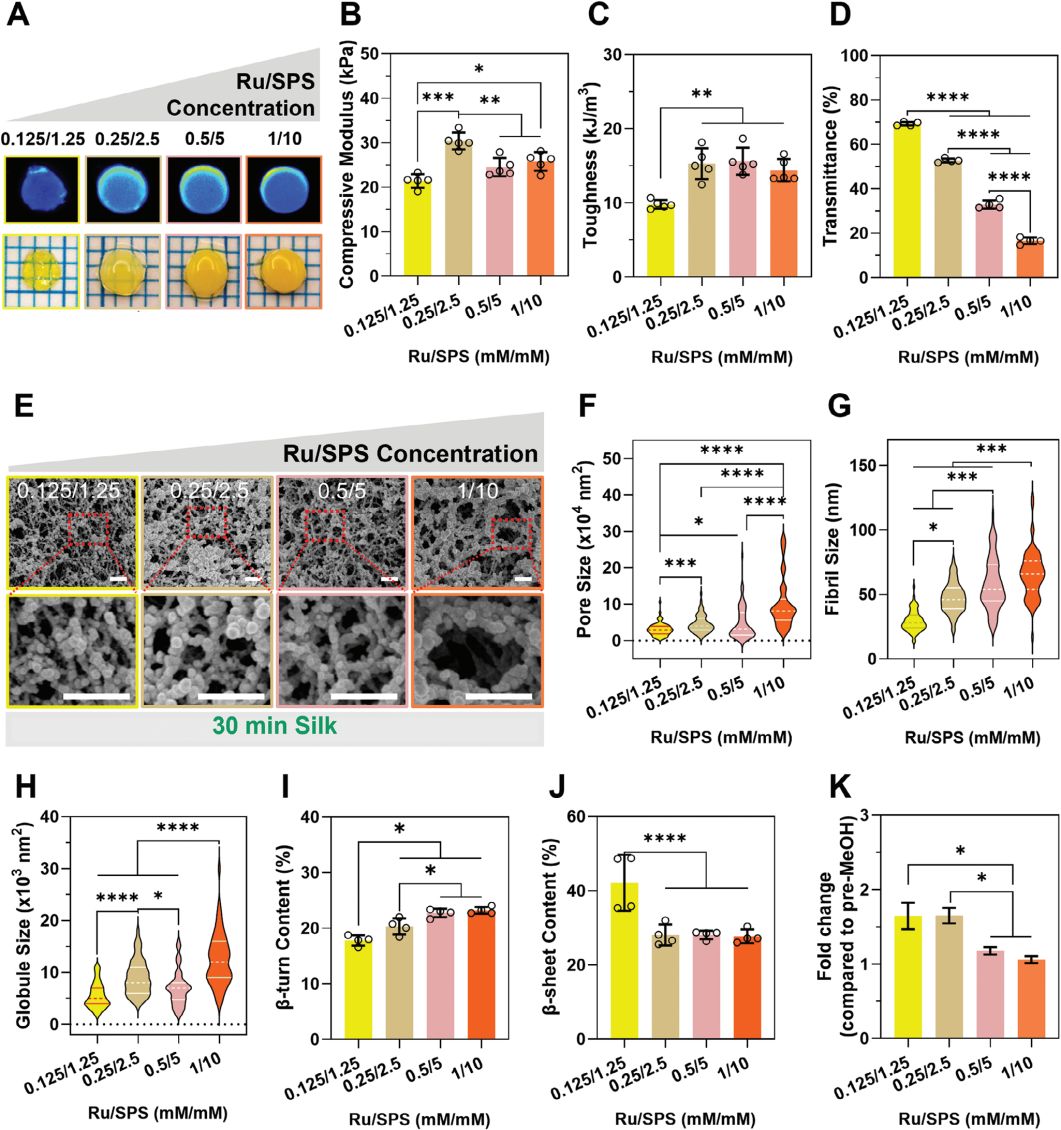
Ru/SPS concentration affects silk self‐assembly and di‐tyrosine crosslinking. 2% silk of medium molecular weight was crosslinked with different Ru/SPS concentrations. A) Representative images of silk hydrogels and their di‐tyrosine fluorescence. B) Compressive modulus and C) Toughness of silk hydrogels. D) Light transmittance through silk hydrogels. E) Representative SEM images of silk hydrogels and analysis of the F) Pore size, G) Fibril diameter, and H) Globule size. *N* = 3–4 with at least 18 counts per image, *n* ≥ 54. Data was collected through a randomized process at the intersections of a grid overlayed over each image. The white scale bars in (E) are 500 nm. I,J) Quantitative analysis of (I) beta‐turn and (J) beta‐sheet contents from deconvolution of FTIR spectrum in the Amide I region. K) Fold change in compressive strength of silk hydrogels after incubation in methanol (MeOH) compared to pre‐MeOH. All data are mean of at least 4 replicates ± SD, *n* = 3–4. *p < 0.05, **p < 0.01, ****p < 0.0001, ns or no indication = no significant difference.

While the standard crosslinking concentration is 0.5/5 mm Ru/SPS, silk hydrogels can be successfully produced from 2% silk using both lower and higher concentrations of Ru/SPS, forming di‐tyrosine bonds (**Figure** **5**A). Macromer fraction analysis indicates that the reaction expelled water and concentrated silk to the same extent irrespective of the Ru/SPS concentration and efficiently incorporated silk into hydrogels, yielding a low (≈5%) sol fraction across all conditions (Figure S6, Supporting Information).

Hydrogels at 0.125/1.25 mm Ru/SPS were softer (Figure 5B), less tough (Figure 5C), and were more difficult to handle than those at higher concentrations. These hydrogels showed a higher fraction of free tyrosines (Figure S6E, Supporting Information), suggesting they were not converted into di‐tyrosine bonds as effectively as with higher Ru/SPS concentrations. From Ru/SPS concentrations of 0.25/2.5 mm and higher, the hydrogel networks exhibited similar mechanical properties (Figure 5B,C). However, the Ru/SPS concentration significantly affected the hydrogel opacity, with higher crosslinker concentrations resulting in less light transmission through the hydrogels (Figure 5D). This suggests that despite similarities in the mechanical properties, the polymer network differed in hydrogels at different Ru/SPS concentrations. This is evident in SEM images (Figure 5E–H), beta‐turn (Figure 5I) and the beta‐sheet crystallinity (Figures 5J and S3B, Supporting Information) of the as‐made hydrogels. At low Ru/SPS concentrations, the polymer spheres, fibers, and pores were smaller than at high crosslinker concentrations. The network of the hydrogels at low Ru/SPS concentrations at 2% silk was akin to that of high‐concentration silk (6%) at higher crosslinker concentrations. This trend is further supported by the observation that the percentage of beta‐turn, which indicated the 180° turning direction of the protein chain, increased with increasing Ru/SPS concentration (Figure 5I), suggesting that crosslinker affects silk self‐assembly. The mechanism behind this effect is not well understood, but one potential explanation involves the generation of SO_4_^2−^ ions during the Ru/SPS reaction, known to cause protein salting out to a greater extent than Na^+^ or HPO_4_^2−^,^[^ ^29^, ^46^ ^]^ which plays an important role in silk self‐assembly (Figure 4).

To investigate the potential for dynamic changes in silk hydrogels using different Ru/SPS concentrations, hydrogel stiffness was determined pre‐ and post‐exposure to methanol. Hydrogels at higher Ru/SPS concentrations had less propensity for beta‐sheet formation, suggesting they are less likely to stiffen over time under physiological conditions (Figure 5K).

**Figure 6:**
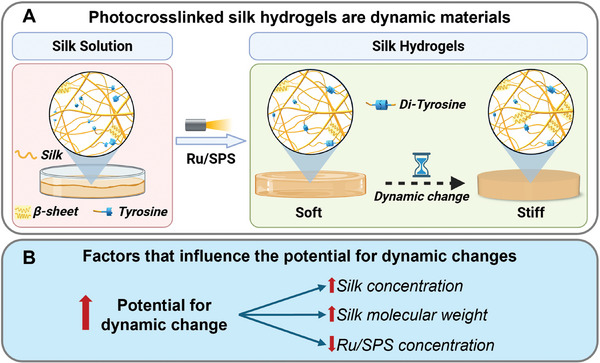
Schematic summary of the observed interplay between silk self‐assembly and di‐tyrosine bond formation and their effect on silk hydrogel formation and dynamic changes in the hydrogel properties over time. A) The interplay between self‐assembly and di‐tyrosine crosslinking in silk hydrogels. B) Factors that regulate the propensity for dynamic changes post‐crosslinking.

Thus, across all the conditions investigated (silk concentration, molecular weight, ions, Ru/SPS concentration), low beta‐sheet crystallinity in the as‐made hydrogels correlated with a lower propensity for dynamic stiffening after di‐tyrosine bond formation. This observation suggested that high flexibility (low silk concentration), high mobility (low molecular weight) of silk protein, high crosslinking density (high photoinitiators concentration), or more ion interactions resulted in more stable hydrogel networks. The phenomenon has also been seen in a dual crosslinked network of silk inks, where silk was partially pre‐photo‐crosslinked for a short time (low mobility and flexibility) with the shear stress of extrusion printing triggering the self‐assembly before being fully crosslinked in HRP that results in more dynamic changes.^[^ ^47^ ^]^ Additionally, increasing crosslinking density (high photoinitiators/crosslinker concentration) could reduce the dynamic changes post‐crosslinking as shown in recombinant protein platforms.^[^ ^48^, ^49^ ^]^ We propose that these observations stem from the reaction kinetics (which is affected by the silk properties, Ru/SPS concentration, and solvents), where early silk self‐assembly affects reaction kinetics (though noting the gelation happens extremely fast in these systems) of covalent crosslinking (Figure S7, Supporting Information), making di‐tyrosine bond formation less effective and ultimately resulting in more dynamic polymer networks (**Figure** **6**). Silk hydrogels made via the Ru/SPS crosslinking system can be considered dual network hydrogels with covalent bonds via tyrosine crosslinks and a beta‐sheet network through hydrogen bonding, where the two networks can be tuned and affect one another.

### 2.6 Understanding of Di‐Tyrosine Crosslinking and Dynamic Changes in Silk can Inform Development of New and Stimuli‐Responsive Silk Materials

The insights obtained in this study not only enhance our understanding of silk self‐assembly and crosslinking through di‐tyrosine bonds but also shed light on the intricate interplay between self‐assembly and crosslinking, paving the way for the development of advanced materials with potential applications in 4D printing, sensing, in vitro models of tissues, drug delivery, and robotics.

**Figure 7:**
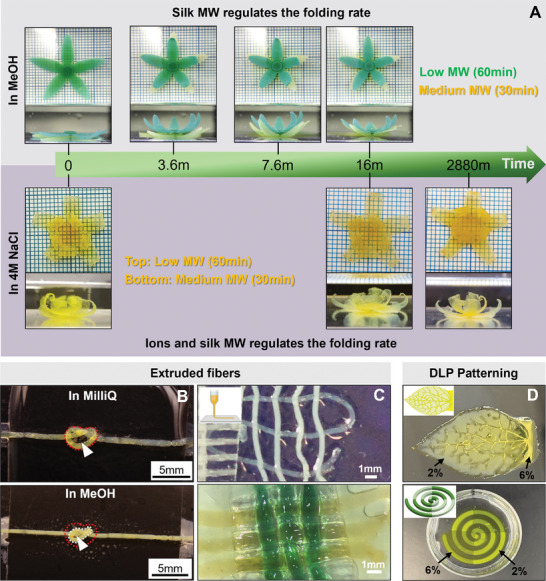
Utilizing dynamic hydrogels for developing novel materials and stimuli‐responsive materials. A) Demonstration of the effect of silk molecular weight on stimuli‐responsiveness. Silk hydrogels were cast into a flower shape with petals made of hydrogels with low (60‐min degumming) or medium (30‐min degumming) molecular weight. The images show different rates of petal folding in response to methanol (top panel) and NaCl (bottom panel). B,C) Representative images showing the feasibility of extruding silk hydrogel fibers and their manipulation through knotting and weaving. (B) The loose silk knot self‐tightened when incubated in methanol. The white arrows indicate the self‐tightening knot of silk fiber due to the stiffening process. The red dashed lines indicate the initial shape of a loose knot, and the white dashed line indicates the shape of a tightened knot. (C) Top panel: Opaque silk fibers are 2% silk hydrogels, and transparent are 6% silk hydrogels. Bottom panel: all silk fibers are made using 6% silk, blue dye was added to one of the solutions to increase the contrast. D) Representative image of 3D patterned leaf and the spiral of 30‐min degumming silk demonstrating silk hydrogel patterning via lithography‐based techniques. Different silk concentrations resulted in silk patterns with different transparency. The transparent parts are 6% wt/v silk, while the opaque parts are 2% wt/v silk.

As proof‐of‐principle, we demonstrated that by adjusting the concentration or molecular weight of silk and employing Ru/SPS crosslinking, we could engineer materials exhibiting dynamic and distinctive contraction and folding in response to an external buffer, illustrated here using 80% v/v methanol and 4 m NaCl. This was evidenced by the folding of petals in flowers crafted from silk hydrogels (**Figures** **7**A and S8 and Videos S1 and S2, Supporting Information) and in the formation of self‐tightening knots from an extruded silk fiber (Figure 7B; Video S3, Supporting Information). Furthermore, the extruded silk fibers could also be weaved at the micro (top panel) or macro scale (bottom panel) (Figure 7C). These insights can also be applied to patterning materials with different properties, as demonstrated here by creating a leaf structure featuring different opacities and mechanical properties patterned in 3D space (Figure 7D).

## 3 Conclusion

The Ru/SPS photo‐crosslinking system facilitated rapid and efficient crosslinking of silk, achieving gelation within seconds of exposure to visible light and incorporating over 90% of silk into the hydrogel. Hydrogels were successfully formed from silk concentrations as low as 1% wt/v. Among the variables tested, silk concentration had the most significant impact on the physical properties of the hydrogels, surpassing the effects of silk molecular weight and crosslinker concentration. These factors collectively influenced the mechanical properties and light transmittance of the hydrogels. A model emerged suggesting that silk self‐assembly occurs both before and after di‐tyrosine bond formation, with silk concentration, molecular weight, and crosslinker concentration affecting phase separation and mesoscale structure formation within the hydrogel network.

Silk concentration also played a crucial role in dynamic changes and hydrogel stiffening over time, with higher concentrations leading to faster and more pronounced stiffening. Ions were found to be influential, particularly at low silk concentrations, resulting in stiffer and tougher hydrogels, while reducing dynamic stiffening over time. A correlation was observed between the initial beta‐sheet content and the propensity for dynamic changes, where higher initial beta‐sheet content (i.e. greater degree of self‐assembly) enabled more dynamic stiffening. This comprehensive understanding opens avenues for developing advanced materials with diverse applications, including 4D biofabrication, sensing technologies, in vitro 3D cancer models, targeted drug delivery systems, and soft robotics.

## 4 Experimental Section

### Materials

*B.mori* silk cocoons were purchased from Sato Yama, Japan. Lithium bromide (LiBr), sodium carbonate (Na_2_CO_3_), sodium persulfate (SPS), Tris(2,2′‐bipyridyl)dichlororuthenium(ii) hexahydrate (Ru), and snakeskin dialysis tubing (3500 MWCO) was purchased from Sigma–Aldrich. Phosphate buffer saline (PBS) tablets and sodium chloride (NaCl) were purchased from Thermo Fisher Scientific.

### Silk Fibroin Isolation

Silk cocoons were processed to generate a regenerated silk fibroin solution as previously described.^[^ ^50^, ^51^, ^52^ ^]^ Briefly, 5 g of cocoons cut into small pieces were boiled in 2 L of 0.02 m Na_2_CO_3_ for 10–60‐min to remove sericin. The degummed fibers (0.2 g mL^−1^ for 10‐min boiled silk and 0.25g mL^−1^ for others) were then dried and dissolved in 9.3 m LiBr for 3 h at 60 °C. LiBr was removed by dialysis against MilliQ water in Snakeskin tubing (3500 MWCO) for 3 days. Regenerated silk fibroin solution was collected and centrifuged two times at 7799 rcf for 15 min at 4 °C to remove the debris and undissolved fibers. The final concentration of collected silk fibroin (referred to as “silk” throughout) solutions was determined through gravimetric analysis by drying a known amount of silk solution and weighing the remaining film. Silk concentration ranged from 7–9% wt/v and silk was stored at 4 °C for use within 4 weeks.

### Silk Hydrogel Preparation

Silk hydrogels were prepared by mixing silk fibroin solution (2–6% wt/v) with Ru/SPS (0.125 mm/1.25 mm, 0.25 mm/2.5 mm, 0.5 mm/5 mm, or 1 mm/10 mm) and irradiated for 3 min using a 30 W lamp (Jobmate, New Zealand) at room temperature. Hydrogels were prepared in MilliQ water, 1xPBS or 0.9% w/v NaCl (final concentration). For most studies (unless specified otherwise), samples (58 µL of silk+Ru/SPS solution) were cast in cylindrical (6 mm diameter, 2.5 mm height) silicone moulds.

### Mass Loss and Swelling Ratio

The initial mass of hydrogels was measured immediately after crosslinking (m_iw_). Three hydrogels of each condition were then frozen and lyophilized to obtain the initial dry mass (m_id_). The macromer fraction (M%) is calculated using Equation (1).

(1) $$M\%=\;\;m_{\mathrm{id}}/m_{\mathrm{iw}}$$

The remaining samples were incubated in MilliQ water at 37 °C for 1 day, and their swollen mass was recorded (m_s_). Samples were then lyophilized to obtain the final dried mass (m_d_). The sol fraction, defined as polymer chains not crosslinked into the network, is calculated using Equation (2).

(2) $$\mathrm{Sol}\mathrm{fraction}=\left[\left(m_{\mathrm{id}}-m_{d}\right)/m_{\mathrm{id}}\right]x100\%$$

The mass swelling ratio (Q) is calculated using Equation (3)

(3) $$Q=\;\;m_{s}/m_{d}$$

### Analysis of the Secondary Protein Structure in Silk Hydrogels

The secondary protein structure in silk hydrogels was studied using Attenuated Total Reflectance Fourier Transform Infrared Spectroscopy (ATR‐FTIR) (PerkinElmer Spectrum Two FT‐IR) on either freshly made samples (after drying overnight at room temperature) or on samples that were incubated overnight in 80%v/v methanol or in MilliQ water, 0.9% w/v NaCl or PBS for 1,7,14, or 28 days. Infrared spectra were collected by averaging 24 scans at a spectral resolution of 4 cm^−1^ within the wavenumber range of 450 to 4000 cm^−1^. Baseline correction was performed for each sample.

The quantification of the secondary structure of silk hydrogels was acquired using a Fourier self‐deconvolution algorithm (FSD) of the IR spectra of the amide I region (1580–1720 cm^−1^). A baseline subtraction was performed on each spectrum in the Amide I region before the fitting process. The fitting procedures were performed using Origin Pro software (OriginLab, USA) employing the Gaussian model. The relative contribution of each secondary structure was determined by integrating the corresponding fitted peaks derived from FSD calculations. The position of each peak was further validated by double differentiating the amide I envelopes with peak assignments as follows:^[^ ^53^, ^54^, ^55^ ^]^ side chains/Tyrosine (1595–1605 cm^−1^); β‐sheets (1610–1635 and 1697–1704 cm^−1^); random coils (1635–1650 cm^−1^); alpha‐helix (1652–1660 cm^−1^), and beta‐turns (1663–1696 cm^−1^). A nonlinear least‐square convergence criterion was used to monitor the error between the original and deconvoluted spectra, ensuring relative errors below 10^−6^. The contribution of β‐sheets and tyrosine was determined by calculating the relative areas of the corresponding peaks in the area‐normalized deconvoluted Amide I profile.

### Optical Properties of Silk Hydrogels

To study the transmittance of light through the silk hydrogels, 100 µL of the silk and Ru/SPS mixture was added per well of a 48‐well plate and irradiated for 3 min with the 30 W desktop lamp (Jobmate, New Zealand). The Ru/SPS solutions without silk were used for background subtraction. The absorbance of hydrogels was measured at 350–800 nm wavelength with a 2 nm step size using a spectrophotometer (SPECTROstar Nano, BMG LABTECH).

### Mechanical Properties of Silk Hydrogels

For compression testing, cylindrical (6 mm diameter, 2.5 mm height) silk hydrogels were compressed to 90% of the initial height at a constant crosshead speed (1 mm s^−1^) at 37 °C in PBS with a 0.1 N pre‐load using a mechanical testing machine with 50N load cell (Instron 5543, USA). Stress–strain curves were obtained, and compressive moduli were determined from the linear region of the stress–strain curves (10–20% strain). Samples were tested freshly made (overnight incubation in PBS), after incubation in 80% v/v methanol (overnight, 4 °C) or after incubation in PBS for 7, 14, 21, or 28 days.

For rheological testing, cylindrical silk hydrogels (8 mm diameter, 4 mm height) incubated overnight in PBS were exposed to a strain sweep from 0.01% to 100% at a constant frequency (1 Hz) with a 3.35 mm gap using Kinexus Pro+ (Netzsch, Germany). The ratio of loss (G″) over storage modulus (G′) was used to determine the linear viscoelasticity regions before the graphs deviated.

To study the crosslinking kinetics,^[^ ^56^, ^57^ ^]^ 2% wt/v or 6% wt/v silk was mixed with Ru/SPS (0.25/2.5, 0.5/5, or 1/10 mm/mm) and loaded on the 25 mm parallel plates of a rheometer (MCR 702e, Anton Paar) set up with the OmniCure S2000 UV light curing system (Excelitas Technologies Corp, USA) and equipped with a UV filter (Rosco IR/UV filter, 400–500 nm, CT, USA). The study was carried out in oscillation mode with a 0.5 mm gap between the parallel plates at 0.1% strain and 1 rad s^−1^ frequency. The measurements were carried out for 5 min, and the light was switched on at 2 min. The storage modulus (G′) was obtained, and the curing rates were calculated based on the slope of the curves within 10s after the light was on. The time derivatives of G′ (dG′/dt) were calculated and the hydrogelation time (Δt_hydrogel_) was determined as the full width at 10% of the maximum value of the peak of the time derivatives of G′ (dG′/dt).^[^ ^57^ ^]^

### Morphological Properties of Silk Hydrogels

Hydrogels were sequentially dehydrated in serial dilutions of ethanol (50%, 70%, 90%, 100%, and 100% v/v) using a Biowave processor (BiowavePro+, Pelco) followed by critical point drying (Autosamdri‐8115, Tousimis and Leica EM CPD300). The dried hydrogels were cut horizontally, mounted onto carbon tape, and coated with platinum (K575X, EMITECH). The samples were imaged at 3 locations using an FEI Nova NanoSEM 230 at 5 kV, probe current of 30 µA. To analyze the pore, fiber, and globule size, at least 3 images of each condition at 40000x magnification were used. An 8×11 grid was overlayed on the image, and the size of the pores, fibers, and globules at the intersecting points of grid lines was measured using the freehand tool of ImageJ (NIH, USA). The data was compiled into violin plots (median and 95% confidence interval).

### Analysis of Responsiveness of Silk Hydrogels to External Stimuli

To demonstrate the potential of silk hydrogels as stimuli‐responsive materials, 2% and 6% wt/v 30‐min and 60‐min boiled silk was mixed with Ru/SPS (0.5/5 mm/mm) and transferred to the 3D polylactic acid‐printed flower mold (Figure S9, Supporting Information), followed by 3 min of irradiation using a 30 W lamp (Jobmate, New Zealand) at room temperature. The cast flowers were transferred to MilliQ water to maintain hydration. The MilliQ water was removed, and solvent (80% v/v methanol or 4 m NaCl) was added. The folding of flower petals was recorded over time (minutes in 80% v/v methanol and days in 4 m NaCl).

The stimuli‐responsive properties were further demonstrated using extruded silk fibers. These fibers were created by loading a mixture comprising 2% or 6% wt/v 30‐min silk and Ru/SPS (0.5/5 mm/mm) into a 1 mL syringe equipped with a 22G blunt needle. The needle was connected to a silicon tube that was lubricated with emulsion oil (6% weight Span‐80: heavy mineral oil). All the syringes, needles, and tubes were covered with aluminum foil except 2–3 cm at the end of the tube. The solution was extruded using a pressure pump at a constant rate (BioX6 or syringe pump), and the light was irradiated continuously near the end of the tube. The extruded silk fibers were collected in a water bath. The size of silk fibers was controlled by the inner diameter of the silicon tube. In this study, tubes with 0.2 and 2 mm inner diameters were used to demonstrate the feasibility of fabricating different sizes of fibers (micro‐ and meso‐scale). The silk fibers could be knotted or weaved, and the self‐tightening response of the loose knot to the external stimuli (80% v/v methanol) was recorded.

The feasibility of 3D (bio)fabricating silk hydrogels was demonstrated using digital light processing (LumenX, CELLINK), where various patterns were projected into silk formulations explored throughout this paper. The hydrogel precursors consisted of silk solution of different concentrations, supplemented with 0.5 mm Ru and 5 mm SPS, as well as 0.5 mg mL^−1^ of tartrazine. Tatrazine was added as a photo‐absorber to enhance pattern precision. All prints were conducted with a 45% projector power level and an exposure time of 2 min. Spatial compartmentalization of different silk hydrogels (2% or 6%) within a construct was further demonstrated using a leaf and spiral design. For the leaf, the stem (6% silk solution) was first patterned, and washed with water to remove uncrosslinked silk. 2% silk solution was then added to the construct to make up the leaf blade, followed with another irradiation step to achieve a complete leaf construct. For the spiral constructs, 2% silk solution was first added to a polystyrene petri dish that is partly masked (top right and bottom left regions covered with Blu Tack), followed by projection of the spiral pattern into the solution from underneath the petri dish. After the first projection, only the uncovered areas (top left and bottom right regions) will allow crosslinking, where a part spiral construct will be formed. The uncrosslinked 2% silk solution was then removed, followed with addition of 6% silk solution to the petri dish. The same spiral pattern was then projected into the petri dish, completing the spiral construct with spatial compartmentalization of 2 types of silk hydrogels at different regions.

### Statistical Analyses

Data are presented as mean ± standard deviation (SD). Statistically significant differences were determined by one‐ or two‐way analysis of variance (ANOVA) and the Tukey post‐test using GraphPad Prism 9 (GraphPad San Diego, CA, USA). Statistical significance was reported at p<0.05 and indicated in the figures as *p < 0.05, **p < 0.01, ***p < 0.001, and ****p < 0.0001, or ns‐ (not significant).

## Conflict of Interest

The authors declare no conflict of interest.

## Supporting information



Supporting Information

Supplemental Video 1

Supplemental Video 2

Supplemental Video 3

## Data Availability

The data that support the findings of this study are available from the corresponding author upon reasonable request.

## References

[1] X. Mu , J. S. K. Yuen , J. Choi , Y. Zhang , P. Cebe , X. Jiang , Y. S. Zhang , D. L. Kaplan , Proc. Natl. Acad. Sci. USA 2022, 119, 2115523119. 10.1073/pnas.2115523119 PMC8795527 35074913

[2] H. A. Tran , T. T. Hoang , A. Maraldo , T. N. Do , D. L. Kaplan , K. S. Lim , J. Rnjak‐Kovacina , Mater. Today 2023, 65, 244.

[3] M. Xie , L. Lian , X. Mu , Z. Luo , C. E. Garciamendez‐Mijares , Z. Zhang , A. López , J. Manríquez , X. Kuang , J. Wu , J. K. Sahoo , F. Z. González , G. Li , G. Tang , S. Maharjan , J. Guo , D. L. Kaplan , Y. S. Zhang , Nat. Commun. 2023, 14, 210. 36639727 10.1038/s41467-023-35807-7 PMC9839706

[4] A. R. Murphy , D. L. Kaplan , J. Mater. Chem. 2009, 19, 6443. 20161439 10.1039/b905802h PMC2790051

[5] J. K. Sahoo , O. Hasturk , T. Falcucci , D. L. Kaplan , Nat. Rev. Chem. 2023, 7, 302. 37165164 10.1038/s41570-023-00486-x

[6] H. Zheng , B. Zuo , J. Mater. Chem. B 2021, 9, 1238. 33406183 10.1039/d0tb02099k

[7] O. C. Onder , S. R. Batool , M. A. Nazeer , Mater. Adv. 2022, 3, 6920.

[8] X. Cui , B. G. Soliman , C. R. Alcala‐Orozco , J. Li , M. A. M. Vis , M. Santos , S. G. Wise , R. Levato , J. Malda , T. B. F. Woodfield , J. Rnjak‐Kovacina , K. S. Lim , Adv. Healthcare Mater. 2020, 9, 1901667. 10.1002/adhm.201901667 31943911

[9] F. Karimi , N. Farbehi , F. Ziaee , K. Lau , M. Monfared , M. Kordanovski , H. Joukhdar , T. G. Molly , R. Nordon , K. A. Kilian , M. H. Stenzel , K. S. Lim , J. Rnjak‐Kovacina , Adv. Funct. Mater. 2024, 34, 2313354.

[10] X. Mu , J. K. Sahoo , P. Cebe , D. L. Kaplan , Polymers 2020, 12, 2936. 33316890 10.3390/polym12122936 PMC7763742

[11] N. R. Raia , B. P. Partlow , M. McGill , E. P. Kimmerling , C. E. Ghezzi , D. L. Kaplan , Biomaterials 2017, 131, 58. 28376366 10.1016/j.biomaterials.2017.03.046 PMC5479139

[12] D. Su , M. Yao , J. Liu , Y. Zhong , X. Chen , Z. Shao , ACS Appl. Mater. Interfaces 2017, 9, 17489. 28470062 10.1021/acsami.7b04623

[13] C. S. Kim , Y. J. Yang , S. Y. Bahn , H. J. Cha , NPG Asia Mater. 2017, 9, e391.

[14] L.‐P. Yan , J. Silva‐Correia , V. P. Ribeiro , V. Miranda‐Gonçalves , C. Correia , A. da Silva Morais , R. A. Sousa , R. M. Reis , A. L. Oliveira , J. M. Oliveira , R. L. Reis , Sci. Rep. 2016, 6, 31037. 27485515 10.1038/srep31037 PMC4971568

[15] V. P. Ribeiro , J. Silva‐Correia , C. Gonçalves , S. Pina , H. Radhouani , T. Montonen , J. Hyttinen , A. Roy , A. L. Oliveira , R. L. Reis , J. M. Oliveira , PLoS One 2018, 13, 0194441. 10.1371/journal.pone.0194441 PMC5884513 29617395

[16] B. P. Partlow , C. W. Hanna , J. Rnjak‐Kovacina , J. E. Moreau , M. B. Applegate , K. A. Burke , B. Marelli , A. N. Mitropoulos , F. G. Omenetto , D. L. Kaplan , Adv. Funct. Mater. 2014, 24, 4615. 25395921 10.1002/adfm.201400526 PMC4225629

[17] A. Maraldo , J. Rnjak‐Kovacina , C. Marquis , Trends Biochem. Sci. 2024, 49, 633. 38653686 10.1016/j.tibs.2024.03.014

[18] J. Rnjak‐Kovacina , L. S. Wray , K. A. Burke , T. Torregrosa , J. M. Golinski , W. Huang , D. L. Kaplan , ACS Biomater. Sci. Eng. 2015, 1, 260. 25984573 10.1021/ab500149p PMC4426347

[19] L. S. Wray , X. Hu , J. Gallego , I. Georgakoudi , F. G. Omenetto , D. Schmidt , D. L. Kaplan , J. Biomed. Mater. Res., Part B 2011, 99B, 89. 10.1002/jbm.b.31875 PMC3418605 21695778

[20] B. P. Partlow , M. B. Applegate , F. G. Omenetto , D. L. Kaplan , ACS Biomater. Sci. Eng. 2016, 2, 2108. 33465886 10.1021/acsbiomaterials.6b00454

[21] M. Baptista , H. Joukhdar , C. R. Alcala‐Orozco , K. Lau , S. Jiang , X. Cui , S. He , F. Tang , C. Heu , T. B. F. Woodfield , K. S. Lim , J. Rnjak‐Kovacina , Biomater. Sci. 2020, 8, 7093. 33079079 10.1039/d0bm01010c

[22] R. Pugliese , M. Montuori , F. Gelain , Nanoscale Adv. 2022, 4, 447. 36132689 10.1039/d1na00688f PMC9418485

[23] E. P. L. Hunter , M. F. Desrosiers , M. G. Simic , Free Radicals Biol. Med. 1989, 6, 581. 10.1016/0891-5849(89)90064-6 2546863

[24] J. K. Sahoo , J. Choi , O. Hasturk , I. Laubach , M. L. Descoteaux , S. Mosurkal , B. Wang , N. Zhang , D. L. Kaplan , Biomater. Sci. 2020, 8, 4176. 32608410 10.1039/d0bm00512f PMC7390697

[25] U.‐J. Kim , J. Park , C. Li , H.‐J. Jin , R. Valluzzi , D. L. Kaplan , Biomacromolecules 2004, 5, 786. 15132662 10.1021/bm0345460

[26] S. P. Zustiak , J. B. Leach , Biotechnol. Bioeng. 2011, 108, 197. 20803477 10.1002/bit.22911 PMC3057087

[27] J. A. Burdick , C. Chung , X. Jia , M. A. Randolph , R. Langer , Biomacromolecules 2005, 6, 386. 15638543 10.1021/bm049508a PMC2678566

[28] K. S. Lim , J. J. Roberts , M.‐H. Alves , L. A. Poole‐Warren , P. J. Martens , J. Appl. Polym. Sci. 2015, 132, 42142.

[29] A. D. Malay , T. Suzuki , T. Katashima , N. Kono , K. Arakawa , K. Numata , Sci. Adv. 2020, 6, eabb6030. 33148640 10.1126/sciadv.abb6030 PMC7673682

[30] L. Lemetti , A. Scacchi , Y. Yin , M. Shen , M. B. Linder , M. Sammalkorpi , A. S. Aranko , Biomacromolecules 2022, 23, 3142. 35796676 10.1021/acs.biomac.2c00179 PMC9364312

[31] P. Zhou , R. Xing , Q. Li , J. Li , C. Yuan , X. Yan , Matter 2023, 6, 1945.

[32] D. Eliaz , S. Paul , D. Benyamin , A. Cernescu , S. R. Cohen , I. Rosenhek‐Goldian , O. Brookstein , M. E. Miali , A. Solomonov , M. Greenblatt , Y. Levy , U. Raviv , A. Barth , U. Shimanovich , Nat. Commun. 2022, 13, 7856. 36543800 10.1038/s41467-022-35505-w PMC9772184

[33] Y. Tu , S. G. Wise , A. S. Weiss , Micron 2010, 41, 268. 19969467 10.1016/j.micron.2009.11.003

[34] S. Ray , T. O. Mason , L. Boyens‐Thiele , A. Farzadfard , J. A. Larsen , R. K. Norrild , N. Jahnke , A. K. Buell , Nat. Chem. 2023, 15, 1306. 37337111 10.1038/s41557-023-01244-8

[35] B. P. Partlow , M. Bagheri , J. L. Harden , D. L. Kaplan , Biomacromolecules 2016, 17, 3570. 27736062 10.1021/acs.biomac.6b01086

[36] B. D. Lawrence , S. Wharram , J. A. Kluge , G. G. Leisk , F. G. Omenetto , M. I. Rosenblatt , D. L. Kaplan , Macromol. Biosci. 2010, 10, 393. 20112237 10.1002/mabi.200900294 PMC3142628

[37] L.‐P. Yan , J. M. Oliveira , A. L. Oliveira , R. L. Reis , J. Tissue Eng. Regener. Med. 2017, 11, 3168. 10.1002/term.2226 27921382

[38] H. I. Okur , J. Hladílková , K. B. Rembert , Y. Cho , J. Heyda , J. Dzubiella , P. S. Cremer , P. Jungwirth , J. Phys. Chem. B 2017, 121, 1997. 28094985 10.1021/acs.jpcb.6b10797

[39] X. Mu , V. Fitzpatrick , D. L. Kaplan , Adv. Healthcare Mater. 2020, 9, 1901552. 10.1002/adhm.201901552 PMC7415583 32109007

[40] F. Hagn , L. Eisoldt , J. G. Hardy , C. Vendrely , M. Coles , T. Scheibel , H. Kessler , Nature 2010, 465, 239. 20463741 10.1038/nature08936

[41] U.‐J. Kim , J. Park , H. Joo Kim , M. Wada , D. L. Kaplan , Biomaterials 2005, 26, 2775. 15585282 10.1016/j.biomaterials.2004.07.044

[42] A. S. Lammel , X. Hu , S.‐H. Park , D. L. Kaplan , T. R. Scheibel , Biomaterials 2010, 31, 4583. 20219241 10.1016/j.biomaterials.2010.02.024 PMC2846964

[43] N. A. Oktaviani , A. Matsugami , F. Hayashi , K. Numata , Chem. Commun. 2019, 55, 9761. 10.1039/c9cc03538a 31355386

[44] R. L. Baldwin , Biophys. J. 1996, 71, 2056. 8889180 10.1016/S0006-3495(96)79404-3 PMC1233672

[45] P. Lo Nostro , B. W. Ninham , Chem. Rev. 2012, 112, 2286. 22251403 10.1021/cr200271j

[46] Y. Zhang , P. S. Cremer , Curr. Opin. Chem. Biol. 2006, 10, 658. 17035073 10.1016/j.cbpa.2006.09.020

[47] F. Agostinacchio , V. Fitzpatrick , S. Dirè , D. L. Kaplan , A. Motta , Bioact. Mater. 2024, 35, 122. 38312518 10.1016/j.bioactmat.2024.01.015 PMC10837071

[48] D. Ding , P. A. Guerette , J. Fu , L. Zhang , S. A. Irvine , A. Miserez , Adv. Mater. 2015, 27, 3953. 26011516 10.1002/adma.201500280

[49] S. H. Hiew , Y. Lu , H. Han , R. A. Gonçalves , S. R. Alfarano , R. Mezzenga , A. N. Parikh , Y. Mu , A. Miserez , J. Am. Chem. Soc. 2023, 145, 3382. 36730942 10.1021/jacs.2c09853

[50] H. Joukhdar , Z. Och , H. Tran , C. Heu , G. M. Vasquez , N. Sultana , M. Stevens , S. Dokos , K. S. Lim , M. S. Lord , J. Rnjak‐Kovacina , Adv. Mater. Technol. 2023, 8, 2201642.

[51] F. Karimi , K. Lau , H. N. Kim , Z. Och , K. S. Lim , J. Whitelock , M. Lord , J. Rnjak‐Kovacina , ACS Appl. Mater. Interfaces 2022, 14, 31551. 35793155 10.1021/acsami.2c03345

[52] K. Lau , C. Heu , M. J. Moore , A. Zhang , B. Akhavan , S. G. Wise , M. M. M. Bilek , M. S. Lord , J. Rnjak‐Kovacina , Mater. Today Adv. 2022, 13, 100212.

[53] X. Hu , D. Kaplan , P. Cebe , Macromolecules 2006, 39, 6161.

[54] A. Barth , Prog. Biophys. Mol. Biol. 2000, 74, 141. 11226511 10.1016/s0079-6107(00)00021-3

[55] Q. Lu , X. Hu , X. Wang , J. A. Kluge , S. Lu , P. Cebe , D. L. Kaplan , Acta Biomater. 2010, 6, 1380. 19874919 10.1016/j.actbio.2009.10.041 PMC2830340

[56] P. Dorishetty , R. Balu , A. Gelmi , J. P. Mata , N. K. Dutta , N. R. Choudhury , Biomacromolecules 2021, 22, 3668. 34460237 10.1021/acs.biomac.1c00250

[57] R. Sun Han Chang , J. C.‐W. Lee , S. Pedron , B. A. C. Harley , S. A. Rogers , Biomacromolecules 2019, 20, 2198. 31046247 10.1021/acs.biomac.9b00116 PMC6765384

