# Supplementary material for: Probing the Interplay of Protein Self‐Assembly and Covalent Bond Formation in Photo‐Crosslinked Silk Fibroin Hydrogels
Source: Small. 2024 Nov 16;21(16):2407923. doi: 10.1002/smll.202407923 (PMC12019910; doi:10.1002/smll.202407923)
Supplement: Supplementary file 1 — Supporting Information [file SMLL-21-2407923-s002.docx]

**Supplementary Figures**


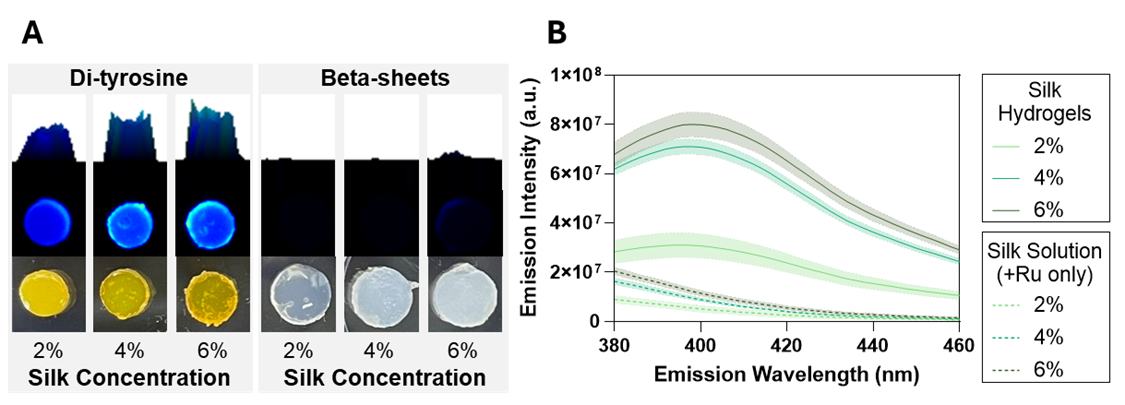


**Figure S1. Di-tyrosine imaging under UV.** **(A)** The typical autofluorescence of di-tyrosine bonds at various silk hydrogel concentrations (left panel) compared to no auto-fluorescence of sonicated beta-sheet induced hydrogels (right panel). **(B)** The fluorescence spectra of silk hydrogel compared to silk solutions in the presence of Ru only. The samples were excited at 280nm and the emission from 380nm to 460nm was obtained using a multi-mode microplate reader (SpectraMax iD5, Molecular Devices).


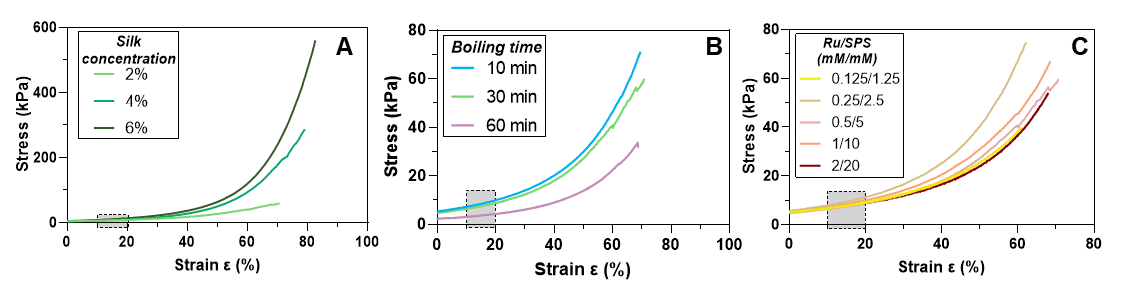


**Figure S2. Stress-strain curves of silk hydrogels at various (A)** silk concentrations, **(B)** silk molecular weight, or **(C)** Ru/SPS concentration.


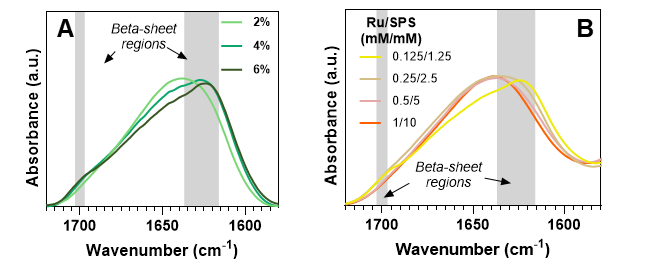

**Figure S3. FT-IR Spectra of Amide I region (1580-1720 cm^-1^) of different silk hydrogels showing the (A)** concentration, or **(B)** Ru/SPS concentration dependent.


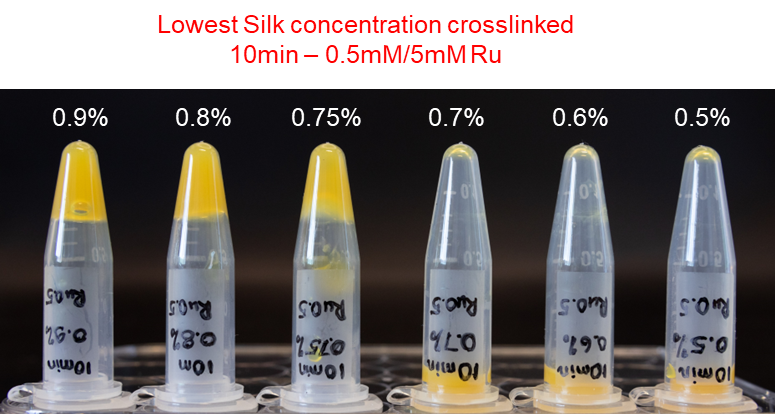


**Figure S4.** **Flipped tubes test showing hydrogels forming at low silk concentrations using high molecular weight.** High molecular weight silk (10-minute degummed) gelled at concentrations of 0.75% wt/wt and above.

**
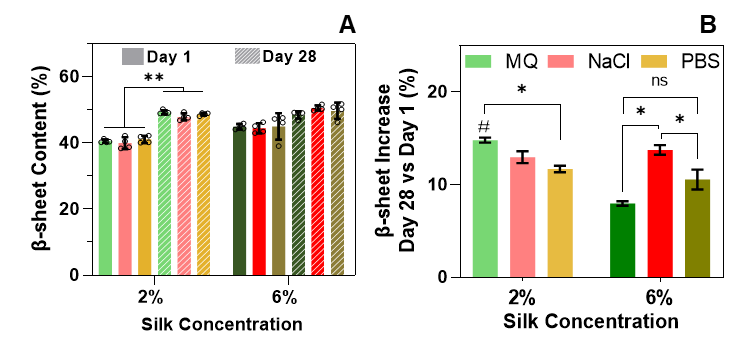

Figure S5. Crystallinity of 2% and 6% silk hydrogels in different solvents over 28 day incubation.** (**A**) Beta-sheet content of silk hydrogels on day 1 and day 28. (**B**) Beta-sheet sincrease at day 28 relative to day 1.


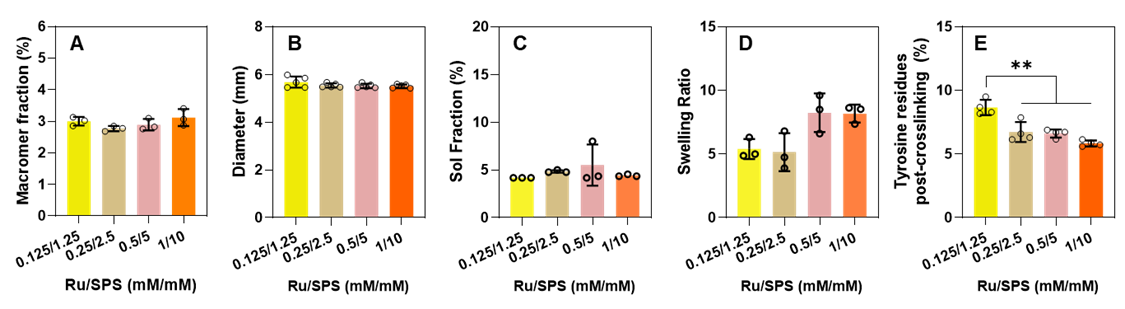


**Figure S6. Ru/SPS concentration regulates crosslinking and hydrogel sizes. (A)** Macromer fraction of silk hydrogels- difference to original is attributed to water expulsion during crosslinking. **(B)** Size of silk hydrogels- difference to the original mmold size is attributed water expulsion during crosslinking. **(C)** Soluble fraction of silk hydrogels showing only 5% uncrosslinked silk. **(D)** Swelling ratio of silk hydrogels increased with increased Ru/SPS. **(E)** Analysis of tyrosine residues from deconvolution of FTIR spectrum in the Amide I region.


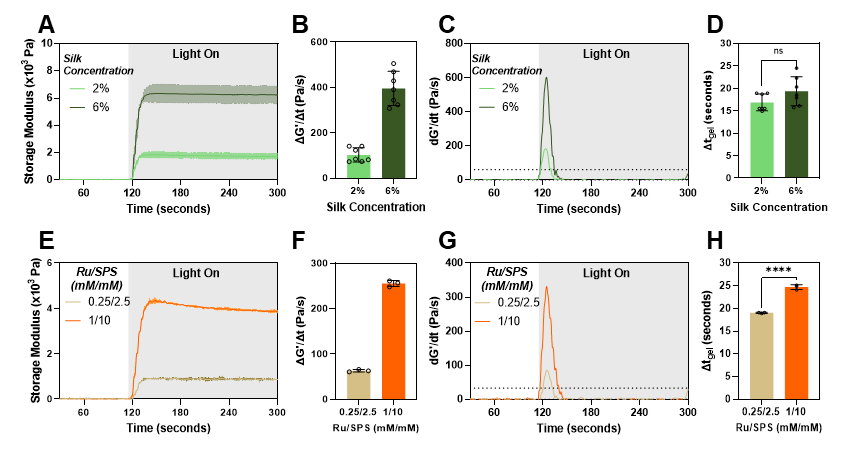


**Figure S7. Kinetic of silk croslinking studied via photorheology.** Storage modulus (G’) of hydrogels at different **(A)** silk concentrations and **(E)** Ru/SPS. The curing rate of the hydrogels at different **(B)** silk concentrations and **(F)** Ru/SPS concentrations were obtained by measuring the slope of the curves within 10 s after turning light on. **(D)**, **(H)** The total hydrogelation time was calculated based on 10% of highest peak of **(C)**, **(G)** the time derivative of storage modulus. **(C-D)** showing the concentration dependent, while **(G-H)** showing the Ru/SPS dependent.


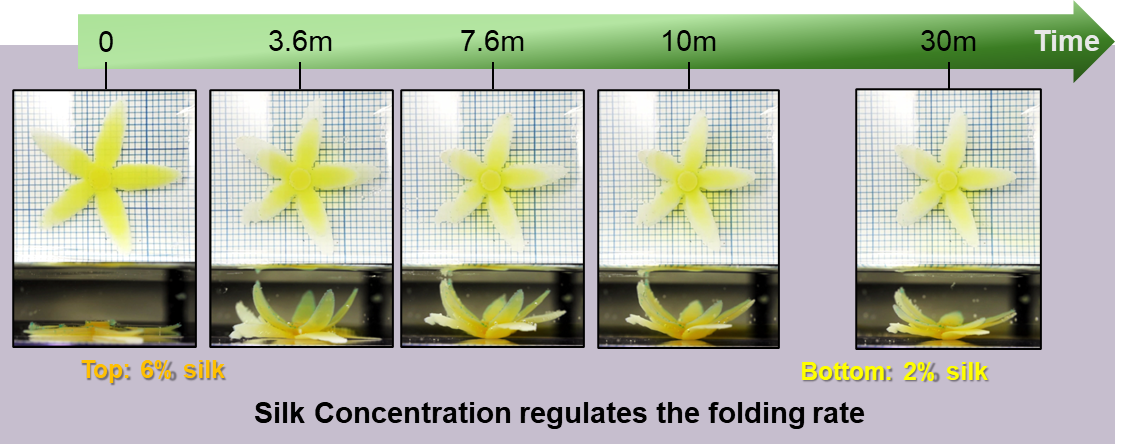


**Figure S8. Silk concentration regulates petal folding rates in 80% MeOH.**


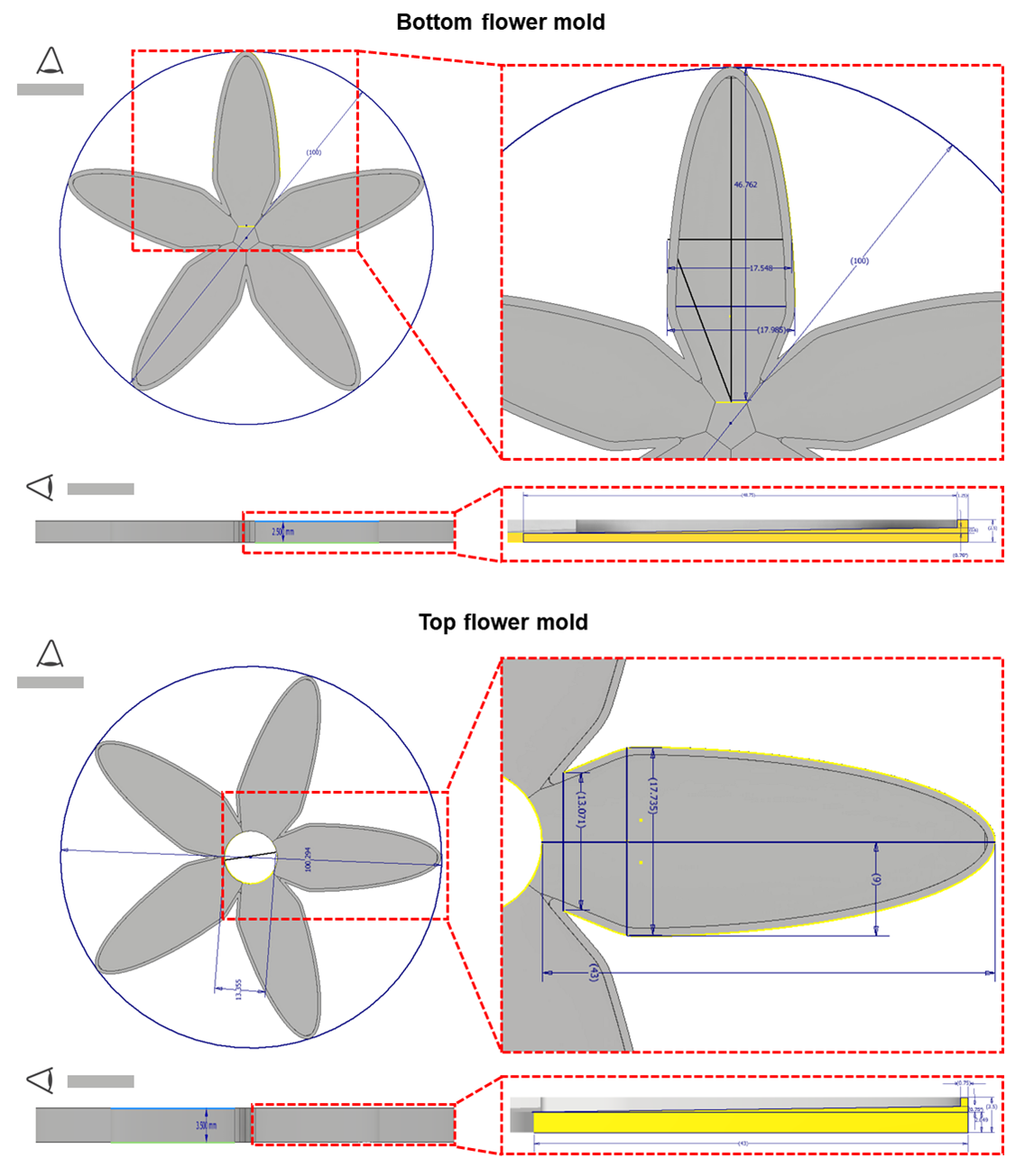

**Figure S9. Design of flower molds in Figure 7.**

**Supplementary Videos**


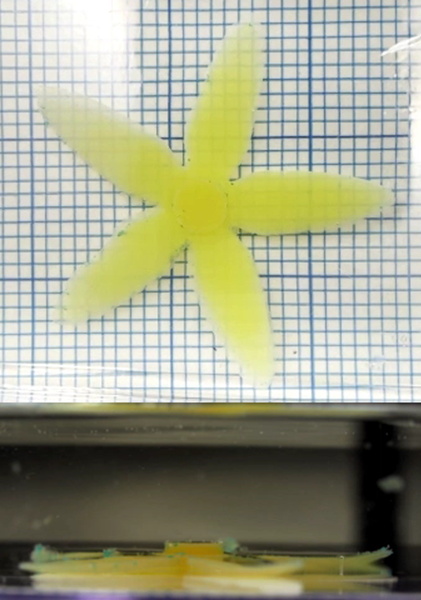


**Supplementary Video 1. Silk concentration controls self-assembly and results in regulating folding rates.** Top cast flower is 6% silk hydrogel, Bottom is 2% silk hydrogel


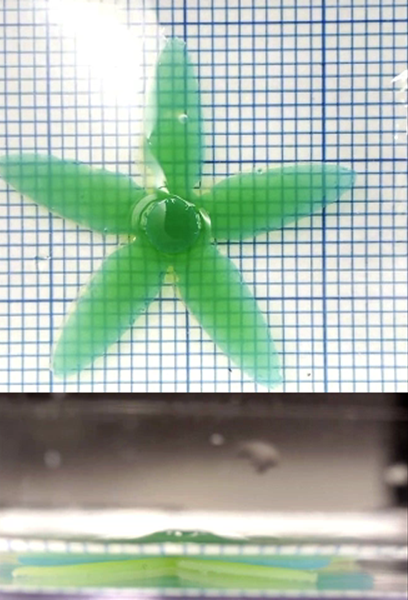


**Supplementary Video 2. Silk molecular weight controls self-assembly and regulates petal folding rates.** Green cast flower is low molecular weight (60min), yellow is medium molecular weight (30min)


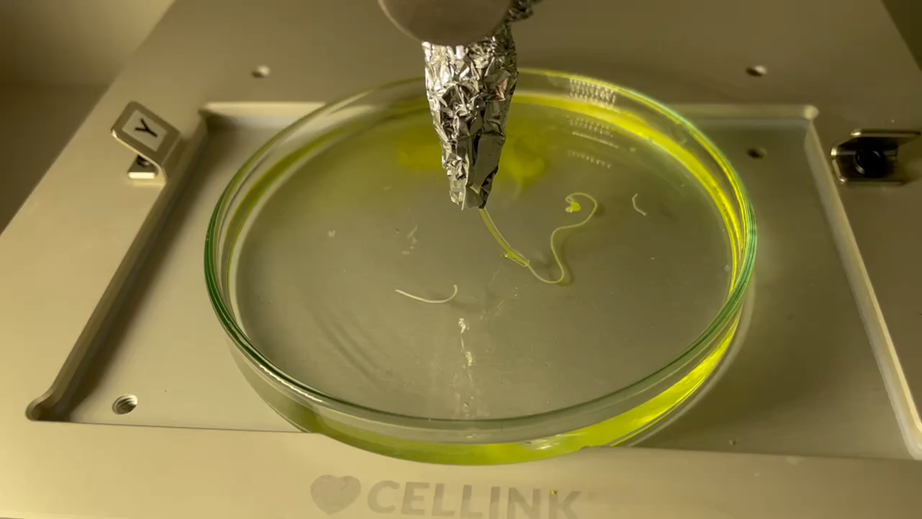


**Supplementary Video 3. Extruded fiber systems using BioX6 with customized printhead.**
